# Supplementary material for: EnzML: multi-label prediction of enzyme classes using InterPro signatures
Source: BMC Bioinformatics. 2012 Apr 25;13:61. doi: 10.1186/1471-2105-13-61 (PMC3483700; doi:10.1186/1471-2105-13-61)
Supplement: Addtional file 5 — The Java code to format the data files, evaluate and predict. The file enzml_java_code.tar.gz contains the Java code used to format database data to ARFF and XML formats, to execute cross and train-test (jackknife) evaluations and to record evaluation results to database. More information is included in the readme.txt file and the Javadoc files. The code can be used with a MySQL database. To use a different database software, other JDBC drivers might be required. [file 1471-2105-13-61-S5.gz › java_code/utils/doc/uk/ac/ed/inf/utils/maputils/OneToManyMap.html]

OneToManyMap


---


|  |  |  |
| --- | --- | --- |
| **Overview** \| **Package** \| **Class** \| **Use** \| **Tree** \| **Deprecated** \| **Index** \| **Help** | |  |
| **PREV CLASS**   **NEXT CLASS** | **FRAMES**    **NO FRAMES**     **All Classes** |  |
| SUMMARY: NESTED \| FIELD \| CONSTR \| METHOD | DETAIL: FIELD \| CONSTR \| METHOD |  |


---


## uk.ac.ed.inf.utils.maputils Class OneToManyMap<T,U>

```
java.lang.Object
  uk.ac.ed.inf.utils.maputils.OneToManyMap<T,U>
```

**Direct Known Subclasses:**: IndexedOneToManyMap

---

``` public class OneToManyMap<T,U> extends java.lang.Object ```

A map containing for each key a set of values. No key can be null. At most
one key can be an empty-string. No value can be null (the Java TreeSet class
used uses "null" as a return message when there is \*no\* mapping for a given
key!). A value can be an empty string.

**Version:**
:   3 Jun 2008

**Author:**
:   Luna De Ferrari luna.deferrari-at-ed.ac.uk

---

| **Field Summary** | |
| --- | --- |
| `java.util.TreeMap<T,java.util.Set<U>>` | `m_map`             the map containing for each key the collection of values that key can have |


**Constructor Summary**

`OneToManyMap()`
  
          Initialise an empty map

`OneToManyMap(java.util.Vector<T> keys,
java.util.Vector<U> values)`
  
          Initialise with a list of key-value pairs


| **Method Summary** | |
| --- | --- |
| `void` | `addAll(OneToManyMap<T,U> mapToAdd)`             Add all elements of a one2many map to another map |
| `boolean` | `addKeyAndValue(T key, U value)`             Add a key-value pair to the map |
| `boolean` | `containsKey(T key)`             Checks if the map contains a key |
| `boolean` | `containsKeyValue(T key, U value)`             Checks if the map contains a specific key-value pair |
| `boolean` | `containsValue(U value)`             Checks if the map contains a value |
| `boolean` | `deleteKeyValue(T key, U value)` |
| `java.util.Set<U>` | `getAKeyValues(T key)`             Get the set of unique values for this key. |
| `java.util.Set<T>` | `getKeys()` |
| `java.util.Vector<java.util.Vector>` | `getKeysVectorValuesVector()`             Get 1. |
| `int` | `getKeyValuesPairsSize()`             Get the number of unique key-value pairs in the map. |
| `java.util.TreeMap<T,java.util.Set<U>>` | `getMap()` |
| `java.util.Set<T>` | `getNonEmptyKeys()`             Get the set of unique, non-empty-string keys for this map. |
| `int` | `getNonEmptyKeyValuesPairsSize()`             Get the number of unique, non-empty key-value pairs in the map. |
| `java.util.Set<U>` | `getNonEmptyValues()`             Get the set of unique, non-empty-string values for this map. |
| `java.util.Set<U>` | `getValues()`             Get the set of unique values for this map. |
| `void` | `initialise()` |
| `boolean` | `mapIsNullOrEmpty()`             public void initialise(Vector keys, Vector values) { this.setMap(); this.generateMap(keys, values); } |
| `int` | `size()` |
| `java.lang.String` | `toCSV()`             Get a list of comma separated [key, value] couples. |
| `java.lang.String` | `toString()` |

**Methods inherited from class java.lang.Object**

`equals, getClass, hashCode, notify, notifyAll, wait, wait, wait`

**Field Detail**

### m\_map

```
public java.util.TreeMap<T,java.util.Set<U>> m_map
```

:   the map containing for each key the collection of values that key can
    have


**Constructor Detail**

### OneToManyMap

```
public OneToManyMap()
```

:   Initialise an empty map

---


### OneToManyMap

```
public OneToManyMap(java.util.Vector<T> keys,
                    java.util.Vector<U> values)
```

:   Initialise with a list of key-value pairs

    **Parameters:**: `keys` - the list of keys: `values` - the list of values (the value for each key in the keys list)


**Method Detail**

### addAll

```
public void addAll(OneToManyMap<T,U> mapToAdd)
```

:   Add all elements of a one2many map to another map

    :   **Parameters:**: `map` - the map whose elements have to be added

---


### addKeyAndValue

```
public boolean addKeyAndValue(T key,
                              U value)
```

:   Add a key-value pair to the map

    :   **Parameters:**: `key` - a key: `value` - a value **Returns:**: true if the value was added, false if the key-value pair already existed

---


### containsKey

```
public boolean containsKey(T key)
```

:   Checks if the map contains a key

    :   **Parameters:**: `key` - **Returns:**: true if the key exists in the map, false otherwise

---


### containsKeyValue

```
public boolean containsKeyValue(T key,
                                U value)
```

:   Checks if the map contains a specific key-value pair

    :   **Parameters:**: `key` -: `value` - **Returns:**: true if the key-value exists, false otherwise (false if the key or value are null)

---


### containsValue

```
public boolean containsValue(U value)
```

:   Checks if the map contains a value

    :   **Parameters:**: `value` - a value **Returns:**: true if the value exists in the map, false otherwise

---


### deleteKeyValue

```
public boolean deleteKeyValue(T key,
                              U value)
```

---


### getAKeyValues

```
public java.util.Set<U> getAKeyValues(T key)
```

:   Get the set of unique values for this key. No values can be null. At most
    one of the values can be an empty string.

    :   **Returns:**: the set of unique values for this key

---


### getKeys

```
public java.util.Set<T> getKeys()
```

---


### getKeysVectorValuesVector

```
public java.util.Vector<java.util.Vector> getKeysVectorValuesVector()
```

:   Get 1. a first vector containing each key as many times as values exist
    for the key. 2. a second vector containing one corresponding value for
    each key entry in the first vector. The two vectors together create a 2
    vectors representation of the map (key in vector 1, value in vector 2).
    Example, for a map: [1:{a, b}, 2:{b, c}] the keys vector will contain:
    [1, 1, 2, 2]. While the values vector will contain: [a, b, b, c]. Read
    together, element by element, they give the map couples.

    :   **Returns:**: a vector of vectors, with the first vector being the keys vector and the second being the values vector.

---


### getKeyValuesPairsSize

```
public int getKeyValuesPairsSize()
```

:   Get the number of unique key-value pairs in the map. No key can be null.
    At most one of the keys can be an empty string. The values can be empty
    strings.

    :   **Returns:**: the number of unique key-value pairs in the map

---


### getMap

```
public java.util.TreeMap<T,java.util.Set<U>> getMap()
```

---


### getNonEmptyKeys

```
public java.util.Set<T> getNonEmptyKeys()
```

:   Get the set of unique, non-empty-string keys for this map.

    :   **Returns:**: the set of unique, non-empty-string keys for this map

---


### getNonEmptyKeyValuesPairsSize

```
public int getNonEmptyKeyValuesPairsSize()
```

:   Get the number of unique, non-empty key-value pairs in the map. No key
    can be null. Empty string keys will not be included, nor will empty
    string values.

    :   **Returns:**: the number of unique key-value pairs in the map

---


### getNonEmptyValues

```
public java.util.Set<U> getNonEmptyValues()
```

:   Get the set of unique, non-empty-string values for this map.

    :   **Returns:**: the set of unique, non-empty-string values for this map

---


### getValues

```
public java.util.Set<U> getValues()
```

:   Get the set of unique values for this map. At most one value can be null.
    At most one value can be an empty string.

    :   **Returns:**: the set of unique values for this map

---


### initialise

```
public void initialise()
```

---


### mapIsNullOrEmpty

```
public boolean mapIsNullOrEmpty()
```

:   public void initialise(Vector keys, Vector values) { this.setMap();
    this.generateMap(keys, values);
    }

---


### size

```
public int size()
```

---


### toCSV

```
public java.lang.String toCSV()
```

:   Get a list of comma separated [key, value] couples.

    :   **Returns:**: a list of comma separated [key, value] couples.

---


### toString

```
public java.lang.String toString()
```

:   **Overrides:**: `toString` in class `java.lang.Object`


---


|  |  |  |
| --- | --- | --- |
| **Overview** \| **Package** \| **Class** \| **Use** \| **Tree** \| **Deprecated** \| **Index** \| **Help** | |  |
| **PREV CLASS**   **NEXT CLASS** | **FRAMES**    **NO FRAMES**     **All Classes** |  |
| SUMMARY: NESTED \| FIELD \| CONSTR \| METHOD | DETAIL: FIELD \| CONSTR \| METHOD |  |


---
